# Sleep deprivation induces genetic damage in mammalian cells: a systematic review

**DOI:** 10.1590/1806-9282.20231097

**Published:** 2024-03-15

**Authors:** Daniel Vitor de Souza, Barbara dos Anjos Rosario, Milena de Barros Viana, Luciana Pellegrini Pisani, Glenda Nicioli da Silva, Daniel Araki Ribeiro

**Affiliations:** 1 Universidade Federal de São Paulo, Institute of Health and Society, Department of Biosciences – Santos (SP), Brazil.; 2 Universidade Federal de Ouro Preto, Laboratory of Clinical Research – Ouro Preto (MG), Brazil.

## INTRODUCTION

Sleep is a natural biological state for reducing wakefulness, metabolism, and motor activity characterized by a reversible state and lack of responsiveness to some stimuli^1,2^. According to the American Academy of Sleep Medicine, the phenomenon can be classified into two stages: non-rapid eye movement (NREM – N1, N2, and N3) sleep stages and rapid eye movement (REM) sleep (R) stage^3^.

Sleep has also been associated with functional brain connectivity and is required for processing information, energy conservation, and restoration^4^. Sleep deprivation occurs when an individual does not sleep well or even insufficient quantity or low quality of sleep, which leads to a decreasing performance and subsequent deterioration in general health^5^. This condition can impair several behavioral and biological activities, affecting cognition and mood, increasing fatigue, and decreasing vigor. This picture impairs speed, decision-making, and accuracy of motor tasks^6^.

Although some environmental factors can interfere with the duration as well as the quality of sleep, it is also genetically controlled^7^. In particular, some studies have demonstrated that sleep deficiency leads to the injury to deoxyribonucleic acid (DNA) in mammalian cells, leading to cellular injury^8-10^. This is consistent with the idea that sleep loss could induce genotoxicity^11^. As a result, this systematic review was motivated to answer the following question: Can sleep deprivation induce genetic damage in mammalian cells?

## METHODS

### Search strategy

In this research, we evaluated genetic damage in mammalian cells induced by sleep deficiency. This systematic review was performed according to the methodology described in the PRISMA guidelines statement^12^. For this purpose, a search was performed on the following scientific databases: PubMed/Medline, Scopus, and Web of Science, and all studies published in the past 10 years (2013–2023) that investigated the relationship between genetic damage and sleep loss were searched. All articles using a combination of the following keywords were selected: "sleep deprivation," "sleep loss," "paradoxical sleep deprivation," "genotoxicity," "genetic damage," "DNA damage," "comet assay," "single-cell gel electrophoresis," "mutation," "sister chromatid exchange," and "micronucleus assay" to refine the search strategy. Boolean operators were used (AND and OR) to combine the descriptors through different combinations as described elsewhere^13^.

### Data extraction

The following data were presented using a particular data collection form: year of study, study design, origin, number of individuals, genotoxicity assay, species used, methodological parameters, negative and positive control groups, blind analysis and statistics, main results, and conclusion.

### Risk of bias in individual studies

The quality assessment of the selected articles was based on previous studies published elsewhere^13^. The following information from the quality instrument was used: (1) study design, (2) identification and treatment of confounding factors, (3) blind analysis, and (4) data analysis. The criteria used to evaluate the study design were the number of participants per group, statistical analysis, and blind analysis. The confounding factors considered were cytotoxicity, number of repetitions, and positive and negative controls. After that, strong, moderate, and weak classifications were used as follows: the study was considered strong when it showed dominance on all items, except one; if it was on two items, it was considered moderate; and if the study did not control three or more items, it was considered weak.

## RESULTS

### Study selection

**Figure 1:**
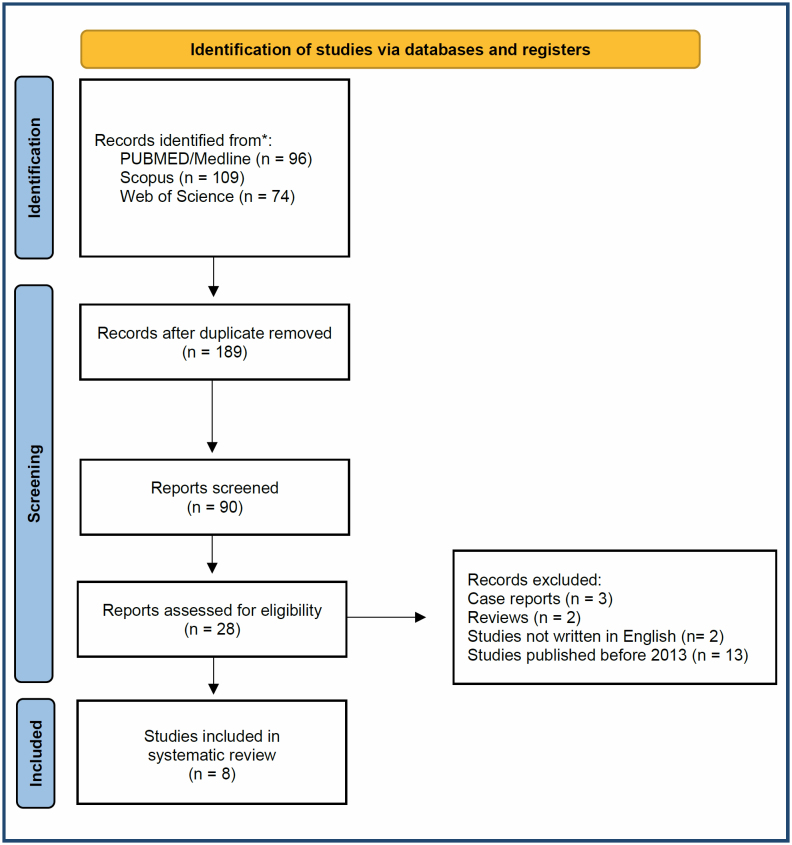
Flowchart of the study.

Initially, the study was able to identify 279 papers, of which 189 publications that were duplicates were excluded from the analysis. After screening all the articles, 161 studies that were not relevant were removed. In addition, reviews, case reports, editorials, papers not written in English, or letters to the editor were not considered. Finally, full texts of the remaining eight studies were sought and thoroughly read by two authors (DVS and DAR). The search strategy is demonstrated in Figure 1.

### Variables related to sleep deprivation and genotoxicity (confounders)

All variables evaluated in the studies are demonstrated in Table 1. The studies evaluated DNA damage by different methodologies. Alkaline single-cell gel (comet) assay was performed in three studies^8-10^. TUNEL assay was applied by Everson et al.^11^, counting cells into slides. Plasma or urine levels of 8-OHdG were evaluated by Everson et al.^11^, Valvassori et al.^12^, and Zou et al.^14^. Zhang et al.^13^ performed FISH using telomere length as a numerical parameter of genotoxicity.

**Table 1:** Variables analyzed in the studies in chronological order.

Author	Target organs	n	Negative control	Positive control	Assay	Number of cells evaluated	Cytotoxicity	Evaluated parameters	Blind analysis	Proper statistics description	Experimental design associated with other conditions
Cheung et al.^10^	Peripheral blood	24 volunteers 9 Males 15 Females 20.08±2.42 years of age	Yes	No	Alkaline comet	100 comets	No	DNA damage %	No	Yes	–
Everson et al.^11^	Liver Lung Heart Jejunum Spleen	Control rats (n=7) Sleep deprivation (n=7–11) Recovery (n=5–6)	Yes	No	TUNEL 8-OHdG	Four sections –	Yes	Counting cells pg 8OHdG/μg DNA	No	Yes	–
Kahan et al.^8^	Skin	12 mice (n=4/group)	Yes	Yes	Alkaline comet	50 comets	No	Tail intensity and tail moment	Yes	Yes	Aging
Moreno-Villanueva et al.^15^	Peripheral blood	16 volunteers 8 Males 7 Females 36.4 ± 7.1 years of age	Yes	Yes	FADU	–	Yes	DNA intensity	No	Yes	Radiation ex vivo
Tenorio et al.^9^	Peripheral blood Heart Kidney Liver Brain	60 rats (n=25/group)	Yes	Yes	Alkaline comet	50 comets	No	Tail intensity	Yes	Yes	Obesity and aging
Valvassori et al.^12^	Brain	40 mice (n=10/group)	Yes	No	8-OHdG	–	No	Plasma concentration	No	Yes	Lithium
Zhang et al.^13^	Lymphocytes Bone marrow Testis	96 volunteers 28 mice (n=7/group)	Yes	Yes	FISH	–	No	Telomere length	No	Yes	Folic acid diet
Zou et al.^14^	Urine samples	16 volunteers	Yes	No	8-OHdG	–	No	Plasma concentration	No	Yes	–

* SD: sleep deprivation; FADU: fluorometric analysis of DNA unwinding; FISH: fluorescence *in situ* hybridization; SCE: sister-chromatid exchange; Dash (–): not applicable.

### Main results

In the study conducted by Tenorio et al.^9^, the genotoxic effect was seen in the peripheral blood, liver, heart, and brain cells of obese old rats submitted to sleep deprivation.

Regarding oxidative DNA damage, 8-OHdG expression was increased in the liver, jejunum, and lung of rats exposed to total sleep deprivation^11^. Similarly, brain cells increased 8-OHdG in mice exposed to paradoxical sleep deprivation^12^. In humans, the same result was observed in urine samples^14^.

The study conducted by Cheung et al.^10^ showed an increase in DNA strand breaks in peripheral blood cells of humans after sleep deprivation. In the study conducted by Zhang et al.^13^, sleep deprivation was associated with telomere shortening in the bone marrow and testis cells of mice and in the peripheral blood cells of humans. Conversely, the studies conducted by Kahan et al.^8^ and Moreno-Villanueva et al.^15^ did not show positive genotoxicity in the blood cells of sleep-deprived humans.

### Assessment of the risk of bias

The quality assessment of manuscripts is shown in Table 2. After reviewing all studies, five papers were classified as strong^8,9,14,15^. In addition, two studies were categorized as moderate at the final rating, because they did not control two relevant variables^11,13^. Finally, two studies were categorized as weak^10,12^.

**Table 2:** Quality assessment and final rating of the studies in chronological order.

Author	Number of confounders	Details	Final rating
Cheung et al.^10^	3	Positive control; cytotoxicity; and blind analysis	Weak
Everson et al.^11^	2	Positive control and blind analysis	Moderate
Kahan et al.^8^	1	Cytotoxicity	Strong
Moreno-Villanueva et al.^15^	1	Blind analysis	Strong
Tenorio et al.^9^	1	Cytotoxicity	Strong
Valvassori et al.^12^	3	Positive control; cytotoxicity; and blind analysis	Weak
Zhang et al.^13^	2	Cytotoxicity and blind analysis	Moderate
Zou et al.^14^	1	Cytotoxicity	Strong

## DISCUSSION

The aim of this study was to evaluate if, and to what extent, sleep deprivation induces genetic injuries in mammalian cells. For this purpose, a total of eight studies were selected in this setting. The single-cell (comet) gel assay is an excellent, reliable method for evaluating DNA strand breakage, including DNA adducts, single- and double-strand breaks, and deficient repair sites. This technique is a simple method that allows the proper investigation of DNA strand breaks that can originate from many contexts and paradigms^16^. In this review, the comet assay was the preferred method for evaluating genetic damage by sleep deprivation as the majority of papers (three studies) have demonstrated positive genotoxicity induced by sleep deprivation in multiple organs of rodents by comet assay. In fact, it has been assumed that DNA damage is driven by sleep^17^. This is because sleep induces nuclear stability, i.e., sleep regulates the homeostatic balance between genetic damage and DNA repair system^17^. Nevertheless, it remains obscure how DNA damage is induced by sleep, and the role of the DNA repair system in this scenario. Anyway, these findings suggest that genetic damage plays an important role as a biological regulator of sleep in mammalian cells^18^. In the past decades, the single-cell gel comet assay Expert Group has established some guidelines for conducting the methodology in a proper way^19^. First, it is mandatory to evaluate at least 25 comets per slide. Additionally, the percentage of the tail (known as tail intensity or % DNA in tail) is the best option when analyzing comet assay associated with an image analysis system.

Furthermore, several studies have demonstrated that sleep deprivation can cause DNA damage using other assays, such as FADU and TUNEL tests. Of particular importance, the studies conducted by Everson et al.^11^ and Valvassori et al.^12^ have demonstrated that sleep deprivation was able to induce oxidative DNA damage, as depicted by 8-OHdG expression. It is important to highlight that 8-OHdG is synthesized from the reaction of the hydroxyl radical (HO•) and guanine, which is the most common way for DNA damage. As a result, a pro-mutagenic agent has been formed when the DNA damage is not repaired^20^.

One important reason that can be categorized as a confounding factor in genotoxicity studies is the adoption of negative and positive controls in the experimental design. For any *in vivo* genotoxicity assay, it is mandatory to demonstrate the specificity as well as the sensitivity of the methodology. Most of the studies included in this review performed tests with positive and negative controls. Nevertheless, the studies conducted by Cheung et al.^10^ and Everson et al.^11^ did not provide concurrent positive control in the experimental design. Another question refers to cytotoxicity. High cytotoxicity is the main confounding factor in genotoxic investigations^21^. Underestimating cytotoxicity may lead to incorrect or misleading data interpretation. In this sense, it is necessary to have more information regarding the association between cytotoxic and genotoxic effects to achieve more sensitive results. Ten studies included in this review did not perform complementary analysis for cytotoxicity.

Considering various parameters used for evaluating the studies included in the review, there is some tendency in the literature showing genotoxic effects that are induced by sleep deprivation. Anyway, such information will bring new insights for a better understanding of the consequences induced by sleep deficiency on genetic material.
